# Post-mortem changes of the vascular system—a thanatological study using multidetector computed tomography

**DOI:** 10.1007/s00414-023-02999-y

**Published:** 2023-04-26

**Authors:** Coraline Egger, Kim Wiskott, Paul Vaucher, Laurent Suppan, Francesco Doenz, Pierre Bize, Silke Grabherr

**Affiliations:** 1 University Center of Legal Medicine Lausanne-Geneva, Rue Michel-Servet 1, 1211 Geneva, Switzerland; 2 Geneva University Hospital and University of Geneva, Geneva, Switzerland; 3 University Center of Legal Medicine Lausanne-Geneva, Chemin de la Vulliette 4, 1000 Lausanne, Switzerland; 4 University of Applied Sciences and Arts Western Switzerland (HES-SO), School of Health Sciences, Boulevard de Pérolles 80, 1700 Fribourg, Switzerland; 5 Division of Emergency Medicine, Department of Anaesthesiology, Clinical Pharmacology, Intensive Care and Emergency Medicine, Geneva University Hospitals, Rue Gabrielle-Perret-Gentil 4, 1205 Genève, Switzerland; 6 Lausanne University Hospital and University of Lausanne, Lausanne, Switzerland; 7 Department of Radiology and Interventional Radiology, Centre Hospitalier Universitaire Vaudois (CHUV), Rue du Bugnon 46, 1011 Lausanne, Switzerland

**Keywords:** Post-mortem MDCT, Post-mortem changes, Thanatology, Vascular system

## Abstract

Forensic pathologists have to deal with post-mortem changes of the human body. Those post-mortem phenomena are familiar and largely described in thanatology. However, knowledge about the influence of post-mortem phenomena on the vascular system is more limited, except for the apparition and development of cadaveric lividity. The introduction of multidetector computed tomography (MDCT) and magnetic resonance imaging (MRI) in the forensic field and the expansion of their usage in medico-legal routine, allow for exploring the inside of corpses differently and may play a part in the understanding of thanatological processes. This study aimed to describe post-mortem changes in the vascular system by investigating the presence of gas and collapsed vessels.

We investigated post-mortem MDCT data of 118 human bodies. Cases with internal/external bleeding or corporal lesion allowing contamination with external air were excluded. Major vessels and heart cavities were systematically explored and a trained radiologist semi-quantitatively assessed the presence of gas.

Collapsed veins were observed in 61.9% of cases (CI95% 52.5 to 70.6) and arteries in 33.1% (CI95% 24.7 to 42.3). Vessels most often affected were for arteries: common iliac (16.1%), abdominal aorta (15.3%), external iliac (13.6%), and for veins: infra-renal vena cava (45.8%), common iliac (22.0%), renal (16.9%), external iliac (16.1%), and supra-renal vena cava (13.6%). Cerebral arteries and veins, coronary arteries, and subclavian vein were unaffected. The presence of collapsed vessels was associated with a minor degree of cadaveric alteration. We observed that arteries and veins follow the same pattern of gas apparition for both the quantity and the location.

In post-mortem radiology, collapsed vessels and intravascular gas are frequently visualized and as a result of all post-mortem changes, the assessment of the distribution of blood can be confusing. Therefore, knowledge of thanatological phenomena is crucial to prevent post-mortem radiological misapprehensions and possible false diagnoses.

## Introduction

Post-mortem changes of the human body are well-known and broadly described in thanatology [1–3]. Decomposition of the body after death involves two parallel processes: autolysis and putrefaction. Autolysis consists of self-dissolution by enzymes released from disintegrating cells, particularly in the pancreas, the stomach, the bowels, and the liver. Putrefaction is due to the action of bacteria and other microorganisms, especially endogenous flora of the respiratory and gastrointestinal tracts, producing gasses (methane, carbon dioxide, hydrogen, ammonia, hydrogen sulfide, and mercaptans). The putrefaction process is significantly influenced by environmental conditions and the prior state of health of the individual (cachexia, adiposity, hypothermia, infectious disease or fever, glycemic level, and blood loss). Nevertheless, there is still little knowledge about the influence of post-mortem reactions on the vascular system, apart from the livor mortis, which is a purplish-blue discoloration of the skin, caused by gravitational forces settling stagnant blood within dilated and toneless vessels.

With the generalization of the use of MDCT (multidetector computed tomography) and MRI (magnetic resonance imaging) in forensic medicine, these imaging modalities now play an essential role in the post-mortem investigations of corpses [4–9]. But they also bring their share of difficulties. Indeed, post-mortem imaging has to deal with artifacts due to post-mortem changes of the body [10]. For example, livor mortis may be mistaken for thrombosis in the vessels because of the hematocrit effect of fluid level [11]. Indeed, hemoconcentration from post-mortem livor mortis appears as intravascular hyperattenuation of the concerned vessels on MDCT, for example in the brain sinuses or large caliber blood vessels such as the aorta. But this phenomenon has also been described within the cardiac cavities. Furthermore, livor mortis may also be misdiagnosed for consolidation from an infectious or tumoral process in the pulmonary parenchyma [11, 12]. And finally, the presence of fluid in body cavities should be differentiated between putrefactive fluid, liquefied fat, and pathologic collection of fluid [11]. Among the most important problems associated with the thanatology of the vascular system, we can list changes in the blood (post-mortem sedimentation and coagulation) [1, 2, 13–15] and changes involving the vascular system itself (presence of intravascular gas and collapsed vessels) [4, 6, 8, 9, 16–28].

This study aimed to describe post-mortem alteration of the vascular system by investigating gas presence and collapsed vessels with post-mortem MDCT.

## Material and method

The results highlighted in this retrospective cross-sectional study come from a database developed for another study that aimed to investigate the incidence and distribution of post-mortem gas detected with MDCT to identify factors that could distinguish artifactual gas from cardiac air embolism [23]. All the selected cases were scanned before the external examination, as is standard practice in our institute. As the prosecutor’s office requested examinations, data collection was planned before the beginning of the study.

### Subjects

Included in this study were a consecutive series of 118 medico-legal cases requiring a medico-legal external examination and submitted to our institute between April 2008 and August 2009. The subjects were deceased without trauma or any invasive medical intervention. No supplementary medico-legal examination was required. Cases were excluded if they presented lesions that imply intracorporeal entrance of external gas or ambient air (e.g., open trauma, gunshot injuries, knife wounds and/or injection stigmata). As no medico-legal autopsy was requested for all the included cases, manners of death were found to be consistent with natural circumstances, suicide by hanging and suicide by absorption of a lethal dose of sodium pentobarbital, based on information received from the police, as well as observations made where the body was found and the medico-legal external examination [23].

Times of death documented on police or medical reports were used to estimate post-mortem intervals (PMI) between death and MDCT examinations. PMI varied approximately from 3 h to 12 days. When the exact time of death was undetermined, it was estimated based on information provided by police investigations (last sign of life (visit, call or any type of conversation), mail in the mailbox, and calendar page at home) and by the external examination of the body (rigor mortis, livor mortis and rectal temperature) [23].

Cases comprised 83 men and 35 women, ranging in age from 20 to 101 years, with an average age of 64.3 years. Fifty-six percent of the cases presented a post-mortem delay of less than 24 h, but a small proportion of the cases (5.9%) had died more than five days previously. Almost half of the cases (43.2%) had a body mass index (BMI) within the standard, less than 10% were underweight, and the others were overweight or obese. Of the 118 cases, sixty-nine died from a natural cause, 49 died from suicide, including 28 by absorption of a lethal dose of pentobarbital of sodium and 21 by hanging (Table 1).

**Table 1:** Characteristics of the 118 selected cases

	Included cases (*N* = 118)	
Gender	Male	83 (70.3%)
	Female	35 (29.7%)
Age (years)	Range (mean)	20–101 (64.3)
	< 20	1 (0.9%)
	21–40	11 (9.3%)
	41–60	33 (28.0%)
	61–80	53 (44.9%)
	> 81	20 (16.9%)
BMI (kg/m^2^)	Range (mean)	13–46 (25.1)
	< 18.5	11 (9.3%)
	18.5–25	51 (43.2%)
	25–30	32 (27.1%)
	30–35	18 (15.3%)
	> 35	6 (5.1%)
Cause of death	Assisted suicide	28 (23.7%)
	Hanging	21 (17.8%)
	Other	69 (58.5%)
Resuscitation	Yes	14 (11.9%)
	No	104 (88.1%)
Post mortem delay (hours)	< 12	31 (26.3%)
	12–24	35 (29.7%)
	24–48	22 (18.6%)
	48–72	16 (13.6%)
	72–120	7 (5.9%)
	> 120	7 (5.9%)

### Quantification of gas and definition and identification of collapsed vessels

Two trained, board-certified radiologists (with at least 10 years of working experience) interpreted the MDCT data. To ensure consistent evaluations between the two radiologists, test cases were evaluated by both radiologists, and the first investigator transcribed results. The investigators discussed their observations during the test cases to reach a unanimous consensus. The amount of gas in the vessels and the heart cavities was semi-quantitatively assessed. The radiologists noticed if vessels could not be identified (N) or had collapsed (C). Their observations were recorded in a table of selected vessels, including 26 arteries and 28 veins, as well as the four heart cavities (Table 2).

**Table 2:** Exhaustive list of the selected structures investigated (the four heart cavities, arteries (*N* = 26) and veins (*N* = 28))

Heart cavities	
Left atrium	Right atrium
Left ventricle	Right ventricle
Selected arteries	Selected veins
Cerebral arteries	Cerebral veins and venous sinuses
Right common carotid artery	Right internal jugular vein
Left common carotid artery	Left internal jugular vein
Right subclavian artery	Right subclavian vein
Left subclavian artery	Left subclavian vein
Brachio-cephalic trunk	Left innominate trunk
Aortic arch	Superior vena cava
Ascending thoracic aorta	Pulmonary trunk
Right coronary artery	Right pulmonary artery
Left coronary artery	Left pulmonary artery
Descending thoracic aorta	Inferior vena cava (supra-renal)
Celiac trunk	Right hepatic vein
Splenic artery	Middle hepatic vein
Superior mesenteric artery	Left hepatic vein
Right renal artery	Portal spaces and veins
Left renal artery	Right renal vein
Inferior mesenteric artery	Left renal vein
Abdominal aorta	Inferior vena cava (infra-renal)
Right common iliac artery	Right common iliac vein
Left common iliac artery	Left common iliac vein
Right internal iliac artery	Right internal iliac vein
Left internal iliac artery	Left internal iliac vein
Right external iliac artery	Right external iliac vein
Left external iliac artery	Left external iliac vein
Right femoral artery	Right femoral vein
Left femoral artery	Left femoral vein

**Fig. 1:**
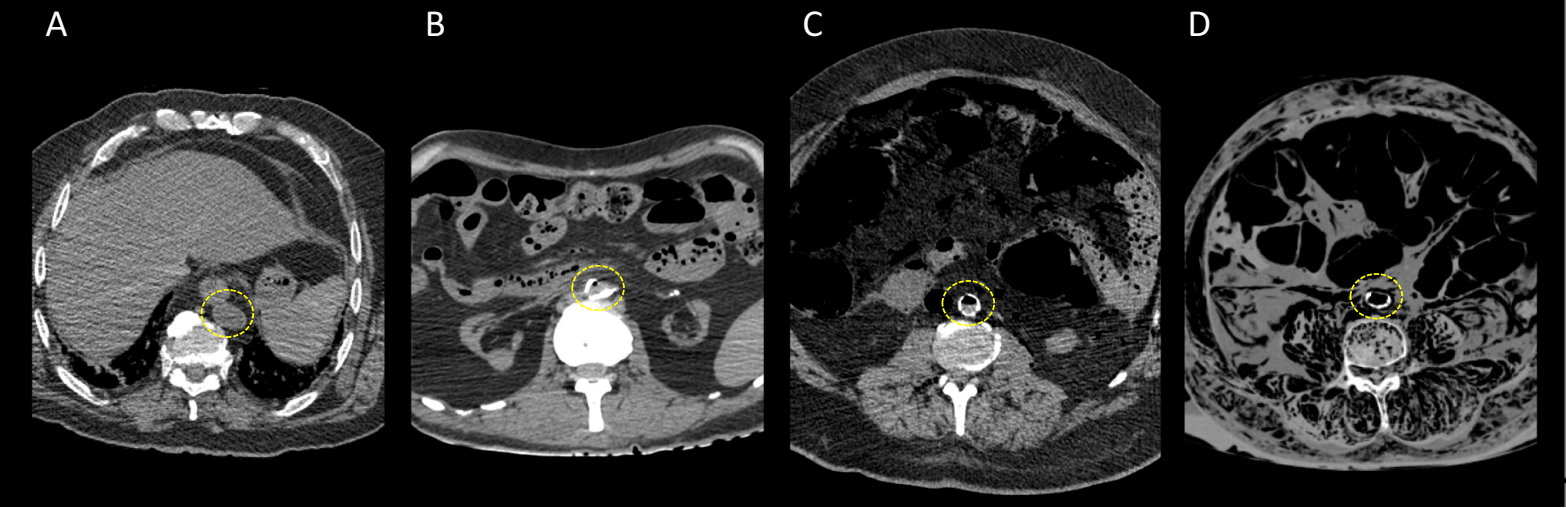
Axial views of a MDCT of the abdomen in parenchymal window with the visualization of the aorta showing a gas grade 0 (circle in **A**), a gas grade I (circle in **B**), a gas grade II (circle in **C**) and a gas grade III (circle in **D**)

**Fig. 2:**
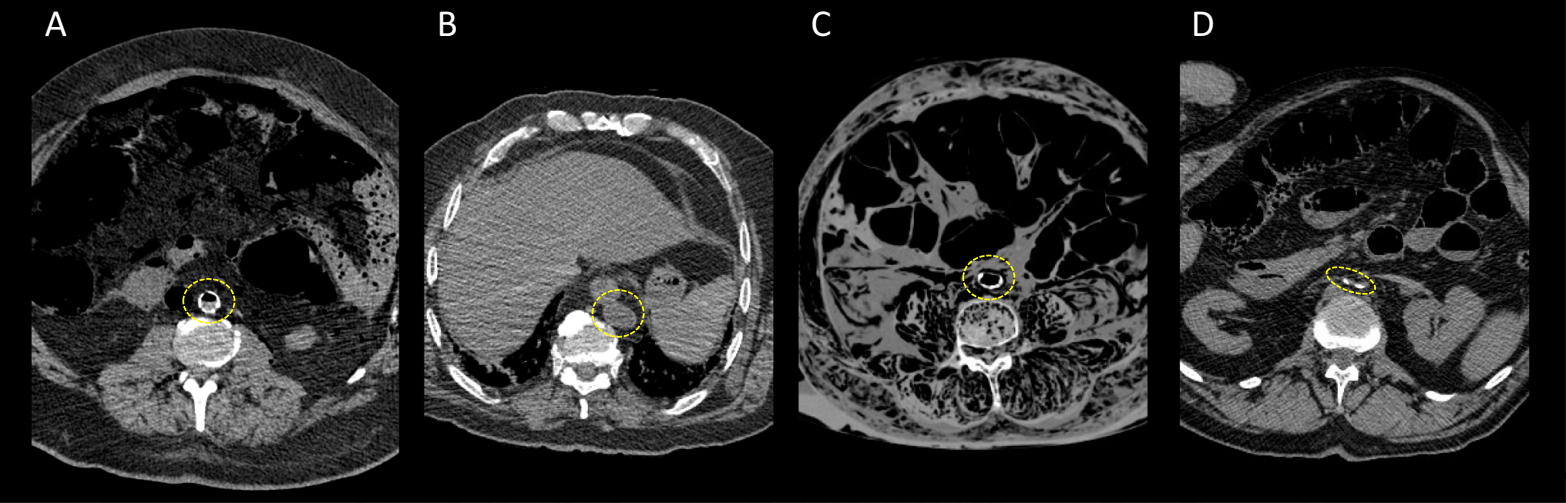
Axial views of a MDCT of the abdomen in parenchymal window with the visualization of a round-shaped aorta (circle in **A**), two examples of an oval-shaped aorta (circles in **B** and **C**) and a collapsed aorta with completely narrowed vascular walls (circle in **D**). The same goes for arteries and veins

The presence of gas in the vessels and heart cavities was graded as either I (one to a few gas bubbles), II (vessel partly filled with gas), or III (vessel completely filled with gas) [23] (Fig. 1). The vessels were considered as collapsed (C) not only when vascular walls were completely narrowed, but also when the lumen of the vessel has lost its rounded shape for a somewhat oval shape (Fig. 2). The heart cavities were considered as collapsed (C) when walls were completely narrowed.

Finally, the radiological alteration index (RAI) was calculated for each case according to Egger et al. [24]. The RAI is a method enabling to radiologically determine the alteration state of cadavers by analyzing the presence of gas from seven anatomical sites (heart cavities, liver parenchyma and vessels, left innominate vena, abdominal aorta, kidneys parenchyma, and vertebra L3).

Statistical analyses then correlated the difference of the vascular collapse to the epidemiological data (age, BMI) and the state of cadaveric alteration and cause of death.

### MDCT

MDCT scans of the selected cases were performed in the supine position with a LightSpeed Ultra 8-row MDCT from General Electric. First, scouts of the entire body in frontal and lateral views were performed with a tube voltage of 120 kV and 10 mA. Then, each scan was conducted in two sets; the first set included the head and neck, and the second set included the arms, thorax, abdomen, and legs (down to the proximal half of the thigh). Technical parameters of the first set were axial scan type with a slice thickness of 1.25 mm, an interval of reconstruction of 1.25 mm, a detector configuration of 8 × 1.25 mm, a beam collimation of 10 mm, a tube voltage of 120 kV and 200 mA, a rotation time of 2 s with full length and eight images per rotation, and a head scan field of view (25 cm maximum). Reconstructions were made using soft and bone filters. Technical parameters of the second set were helical scan type with a slice thickness of 1.25 mm, an interval of reconstruction of 1 mm, a detector configuration of 8 × 1.25 mm, a beam collimation of 10 mm, a tube voltage of 120 kV and 300 mA, a rotation time of 0.8 s with full length and 13.5 mm per rotation, a large scan field of view (50 cm maximum), and a pitch of 1.35:1. Reconstructions were made using standard and bone filters [23].

### Data analysis

Spearman’s Rho and Kruskall Wallis non-parametric tests were used for statistical analyzes that were performed using Stata® intercooled version 16 (StataCorp, College Station, TX, USA). A *p* value < 0.05 was considered statistically significant.

## Results

### Generalities

In all structures combined, two-thirds of cases presented at least one area of collapse (arterial, venous, or cardiac cavities). At the arterial level, only 31% of cases showed a collapse compared to the veins, where more than 60% collapsed. In a few cases (6.8%), cardiac cavities collapsed (Table 3).

**Table 3:** Presence or absence of collapse depending of the structure (arteries, veins and cardiac cavities)

	Presence of collapsus	Absence of collapsus
Arterial	39 (33.1)	79 (66.9)
Vein	73 (61.9)	45 (38.1)
Cardiac cavities	8 (6,8)	110 (93.2)
All vessels	77 (65.3)	41 (34.7)

Collapsed veins were observed in 61.9% of cases (CI95% 52.5 to 70.6) and arteries in 33.1% (CI95% 24.7 to 42.3). Vessels most often affected were for arteries: common iliac (16.1%), abdominal aorta (15.3%), external iliac (13.6%), and for veins: infra-renal vena cava (45.8%), common iliac (22.0%), renal (16.9%), external iliac (16.1%), and supra-renal vena cava (13.6%). Cerebral arteries and veins, coronary arteries, and subclavian vein were not affected.

There was a significant negative association (*p* = 0.016) between the number of collapsed vessels and increased age (Spearman’s Rho = −0.222).

### Collapse and cadaveric alteration

We paired vessels and studied the odds of having a collapsed artery in the presence of a collapsed corresponding vein. We observed that arteries and veins follow the same pattern of gas apparition for both the quantity and the location. Indeed, the odds of having a collapsed artery increased by 16.6 (CI95% 11.4 to 24.3) when the paired vein collapsed. Furthermore, if at least one of the two vessels collapsed (*n* = 188), so was the other in 38.8% of the pairs.

The total number of collapsed vessels was correlated to the radiological alteration index [23] (Spearman’s Rho = 0.382; *p* < 0.0001), and we observed that the presence of collapsed vessels was associated with a minor degree of cadaveric alteration. Moreover, the presence of gas in a vessel (16.1%) increased by two (CI95% 1.6 to 2.5) the odds of finding a collapse in the same vessel (6.0%). The probability of a collapsed vessel also depended on the post-mortem delay (Spearman’s Rho = 0.399; *p* < 0.0001).

### Collapse and body mass index

The correlation between BMI and the collapse of blood vessels was also explored. The collapse of blood vessels was not associated with BMI and no correlation was found between being overweight and collapse (Kruskal-Wallis test, *p* = 0.64).

### Collapse and cause of death

There was a statistically significant difference in the probability of presenting a collapse (arterial, venous, or cardiac) according to the cause of death. A natural cause of death showed the most significant probability of having a vessel collapse (Kruskal-Wallis test, *p* = 0.0216).

## Discussion

This study aimed to describe post-mortem alteration of the vascular system by investigating gas presence and collapsed vessels with post-mortem MDCT.

We observed that two-thirds of cases presented at least one area of collapse. Some authors postulate that in case of fatal hemorrhage, the collapse of major vessels allows quantification of the volume of blood remaining in the body, using measurements of cross-sectional areas of major blood vessels such as the aorta or the inferior vena cava, both in a post-mortem [25] and in a clinical setting [26, 27]. Though this phenomenon has been mentioned since the late 80 s in clinical radiology as the “hypoperfusion complex”, involving the association of hypovolemia and hypotension with collapsed inferior vena cava on MDCT [26, 27, 29, 30], the correlation between clinical and post-mortem MDCT findings is difficult to make, mainly due to the cessation of blood flow and the resulting modifications of hemodynamic conditions. Moreover, vena cava collapse is a sign of relative or absolute hypovolemia sought by ultrasound by emergency physicians. Still, it is an unreliable method to predict the fluid administration responsiveness of patients during resuscitation [31]. Furthermore, although we observed that arteries and veins follow the same pattern of gas apparition, for both the quantity and the location, we also noted that the number of collapsed veins is twice the number of arteries. One explanation for this phenomenon is the difference in the structure of the wall of the vessels. Veins have a constitutionally more flexible wall than arteries. Although arteries may sometimes present pathological processes, such as atherosclerosis decreasing the elasticity of the wall, healthy arteries have a thicker and more rigid wall than veins. However, we do not have a definite explanation for the fact that cerebral arteries and veins, coronary arteries, and subclavian vein were not affected by the phenomenon of collapse, except for the fact that cerebral arteries and veins, as well as coronary arteries, are quite small vessels and that the phenomenon could have escaped the eye of observers.

Concerning the possible association between the presence of gas and a collapsing phenomenon, we found out that the probability of having a collapsed vessel is dependent on the post-mortem delay and that the presence of collapsed vessels was associated with a minor degree of cadaveric alteration. As gas inside the body, particularly in the vessels, is directly related to PMI [23], our observation that the odds of finding a collapse in a vessel are doubled when the vessel in question contains gas is consistent with the current knowledge. Besides, MDCT being very sensitive, it can detect very small amounts of gas, which is impossible to do with conventional autopsy [4–9, 11, 20, 21, 23, 24, 27, 28]. The presence of gas, especially in the heart, is important to highlight for the forensic pathologist, not only as a cause of death but mostly as a vitality sign. However, gas from cadaveric alteration already appears a few hours after death [23]. For this reason, post-mortem radiological diagnosis between vital air embolism and putrefaction gas is difficult to make, nearly impossible without gas sampling and chromatographic analysis [11, 17, 23, 24]. Still, this differential diagnosis is crucial for the forensic pathologist.

We also noticed a significant negative association between the number of collapsed vessels and increased age at death, allowing us to assume that, regarding arteries, atherosclerosis might have an influence by stiffening the wall of the vessels and preventing its collapse. Indeed, according to the scientific clinical literature, atherosclerosis is a disease of aging [32–34]. However, a direct correlation between non-collapsed arteries and atherosclerosis was not performed because the state of arterial sclerosis was not graded on the MDCT data.

Our results also showed that the probability of finding a collapsed vessel is higher when the cause of death is natural. We don’t have any explanation for this result, especially since our data only includes a small portion of unnatural deaths. Nevertheless, we would have hypothesized that the suddenness of death could be a clue. Still, the non-natural death group consisted in persons who committed suicide, whether by hanging or by absorption of pentobarbital of sodium, and those two methods of suicide imply a rather rapid death, in a few minutes, as natural deaths [1–3]. Another hypothesis is that cardiopulmonary resuscitation (CPR) may play a role, considering vascular filling with fluids. Still, our results are against this assumption, with more collapsed vessels in the natural deaths group, whereas this group has a higher rate of CPR.

Post-mortem imaging is known for dealing with many artifacts due to post-mortem changes in the body [10]. Among the most important issues associated with the thanatology of the vascular system, we can list changes in the blood (post-mortem sedimentation and coagulation) [1, 2, 13–15] and changes involving the vascular system itself (presence of intravascular gas and collapsed vessels) [4, 6, 8, 9, 16–28]. Our study confirmed that, in the absence of blood loss or body lesions allowing contamination with external air, the presence of gas in the vascular system and/or collapsed vessels is frequently observed and might be related to normal thanatological processes. Consequently, even in presence of blood loss, we would be cautious about using vascular collapse on post-mortem imaging as a sign of blood loss.

For all these reasons, it is important to be aware of post-mortem changes and the stage of cadaveric alteration to interpret post-mortem MDCT findings properly [11, 23, 24, 28]. Furthermore, our results support that evaluating the vessels on post-mortem native MDCT is complicated, even more, when collapsed, preventing both lumen and wall interpretation. Hence the interest in post-mortem angiography [10, 35, 36]. However, according to Grabherr et al. [37], this technique also required quality criteria to perform a proper radiological interpretation and minimize the risk of radiological misinterpretation with post-mortem perfusion. Indeed, Grabherr et al. [37] developed a standardized technique with a standardized protocol (multiphase post-mortem CT angiography) enabling high-quality post-mortem CT angiography based on the fact that the most important criteria to increase the quality of the radiological examination is that a correct radiological interpretation is directly related to the complete filling of the vascular system with the contrast agent. Therefore, post-mortem CT angiography is the appropriate technique and modality to evaluate the vascular lumen looking for stenosis, occlusions, thrombosis, etc. However, concerning the appreciation of the vascular wall, the best radiological modality seems to be MRI, according to Coolen et al. [38]. But that does not change the fact that if the vessel of interest is collapsed, contrast agent injection is required to perform high-quality vascular diagnosis.

Finally, this study raises more interrogations and forces us to admit that our knowledge on the subject is still speculative. We need to find out where the blood is when collapsed vessels are without blood loss. This issue needs further investigation, and post-mortem imaging techniques may provide clues or the answer.

## Conclusion

Collapsed vessels and intravascular gas are frequently visualized in post-mortem radiology, and, as a result of all post-mortem changes, the assessment of the distribution of blood can be confusing. Therefore, knowledge and understanding of the thanatological phenomenon are crucial to prevent post-mortem radiological misapprehensions and potential false diagnoses.
