# Correction to: Post‑mortem changes of the vascular system—a thanatological study using multidetector computed tomography

**DOI:** 10.1007/s00414-023-03014-0

**Published:** 2023-05-10

**Authors:** Coraline Egger, Kim Wiskott, Paul Vaucher, Laurent Suppan, Francesco Doenz, Pierre Bize, Silke Grabherr

**Affiliations:** 1 University Center of Legal Medicine Lausanne-Geneva, Rue Michel‑Servet 1, 1211 Geneva, Switzerland; 2 Geneva University Hospital and University of Geneva, Geneva, Switzerland; 3 University Center of Legal Medicine Lausanne-Geneva, Chemin de la Vulliette 4, 1000 Lausanne, Switzerland; 4 School of Health Sciences, University of Applied Sciences and Arts Western Switzerland (HES‑SO), Boulevard de Pérolles 80, 1700 Fribourg, Switzerland; 5 Division of Emergency Medicine, Department of Anaesthesiology, Clinical Pharmacology, Intensive Care and Emergency Medicine, Geneva University Hospitals, Rue Gabrielle‑Perret‑Gentil 4, 1205 Genève, Switzerland; 6 Lausanne University Hospital and University of Lausanne, Lausanne, Switzerland; 7 Department of Radiology and Interventional Radiology, Centre Hospitalier Universitaire Vaudois (CHUV), Rue du Bugnon 46, 1011 Lausanne, Switzerland

**Correction to** **:** **International Journal of Legal Medicine (2023)**

**https://doi.org/10.1007/s00414-023-02999-y**

The original article published with inverted author names. Family name was captured first instead of given name. This is now correctly presented here.

The original article has been corrected.

